# Peripheral Neuritis Following Emetine Treatment of Amebic Dysentery

**Published:** 1918-05

**Authors:** 


**Peripheral Neuritis Following Emetine Treatment of Amebic Dysentery.** By KILGORE. Abs. from *China Med. Journal*, May, 1919.

The author reports seven cases, three adults and four children, all of whom developed peripheral neuritis after taking emetine. Muscular weakness, pain (chiefly of the lower extremities). tenderness on pressure, wrist drop and toe drop, hyperesthesia of the soles of the feet, were noted among the symptoms. Experiments made upon dogs suggest that emetine may produce peripheral neuritis in these animals. The children varied in age from 4 to 8 years, and the amount of emetine given to them varied from 4 to 6 grains.

